# Clinical and Safety Outcomes Associated with Extended Treatment of Venous Thromboembolism: A Network Meta-Analysis

**DOI:** 10.3390/jcdd9120414

**Published:** 2022-11-25

**Authors:** Zhiqiang Liu, Jiangshan Tan, Yuanrui Deng, Lu Hua, Tingting Guo

**Affiliations:** Center for Respiratory and Pulmonary Vascular Diseases, Department of Cardiology, Key Laboratory of Pulmonary Vascular Medicine, National Clinical Research Center of Cardiovascular Diseases, National Center for Cardiovascular Diseases, State Key Laboratory of Cardiovascular Disease, Fuwai Hospital, Chinese Academy of Medical Sciences and Peking Union Medical College, Beijing 100037, China

**Keywords:** venous thromboembolism, extended treatment, novel oral anticoagulants

## Abstract

Background: Many anticoagulant strategies are available for the extended treatment of venous thromboembolism, yet little guidance exists regarding which drug is most effective and safe. Aim: A network meta-analysis was performed to resolve this uncertainty. Methods: We searched the medical literature through June 2022 for randomized controlled trials (RCTs) evaluating the efficacy and safety of anticoagulants for adults with VTE compared with other anticoagulants or a placebo. Results: We identified 13 eligible RCTs in 12 articles. All pooled hazard ratios (HR) and 95% credible intervals (CrI) mentioned below, except that for aspirin, were calculated by comparison with standard-intensity warfarin. Novel oral anticoagulants (NOACs) were not inferior to standard-intensity warfarin in preventing recurrence, and edoxaban was ranked first among the NOACs (HR, 0.99; 95% CrI, 0.70–1.39). All the NOACs, except rivaroxaban, were superior to standard-intensity warfarin in preventing bleeding events. Apixaban was ranked first and was considered to be safer than other NOACs for control of both major bleeding (HR = 0.07, 95% CrI: 0.01–0.37) and clinically relevant non-major bleeding (CRNMB, HR = 0.30, 95% CrI: 0.13–0.67). Edoxaban was ranked second among the NOACs for control of major bleeding (HR = 0.44, 95% CI: 0.21–0.88), and dabigatran was ranked second among the NOACs for control of CRNMB (HR = 0.54, 95% CrI: 0.4–0.73). Conclusions: There existed no statistically significant differences in recurrence between NOACs and standard-intensity warfarin, and NOACs were associated with a lower risk of bleeding events. Edoxaban effectively prevented VTE recurrence and major bleeding, and apixaban was the best anticoagulant for controlling bleeding events.

## 1. Introduction

Venous thromboembolism (VTE) comprises deep vein thrombosis (DVT) and pulmonary embolism (PE). The incidence of VTE is more than 900,000 cases per year in the United States and more than 750,000 per year in Europe; approximately one-third of patients with VTE have PE [1], and the incidence rate increases exponentially with age. VTE is the third leading cause of vascular death after myocardial infarction and stroke [2]. Patients with VTE have a high risk of VTE recurrence within the first 3–4 weeks following the index event; therefore, high-intensity anticoagulant therapy and frequent monitoring are needed [3]. It is recommended that patients with VTE receive anticoagulation treatment for at least 3 months because the recurrence risk increases remarkably when the duration is less than 3 months [4].

Extended anticoagulant therapy is defined as an anticoagulation time longer than 3 months and is recommended for patients at high risk of recurrence. VTE can recur after discontinuation, even with a prolonged duration of anticoagulation therapy, and the cumulative recurrence rate sharply increases, matching that in those without extended anticoagulation therapy [5,6]. According to clinicians, the main challenge with prolonged anticoagulant therapy is its efficacy and safety in terms of recurrence prevention and bleeding control. The 2019 European Society of Cardiology (ESC) guidelines recommend that the duration of anticoagulation therapy be decided according to the risk of recurrence and bleeding [7].

Warfarin is a traditional oral anticoagulant for the treatment of VTE and is effective in preventing VTE recurrence, but interactions with various foods and clinical medications [8], frequent and inconvenient laboratory monitoring, and concern about bleeding, often lead to a reluctance to continue warfarin therapy. Although some patients have good compliance, manage their diet, accept regular monitoring of their INR, and receive dose adjustments in a timely manner, achieving stabilized anticoagulation intensity with warfarin remains very difficult [9]. Novel oral anticoagulants (NOACs) do not require laboratory monitoring yet are effective as a single agent for the treatment of acute venous thromboembolism and for continued treatment, with no food interactions and only a few drug interactions. NOACs are gradually replacing warfarin in the anticoagulation of VTE, but their efficacy and safety need further analysis. We conducted a network meta-analysis (NMA) to assess the relative efficacy and safety of different anticoagulants used for the extended treatment of VTE, and to propose recommendations for clinical strategies.

## 2. Methods

We performed a network meta-analysis (NMAs) in accordance with the prespecified and peer-reviewed preferred reporting items for systematic reviews and meta-analyses (PRISMA) extension guideline and checklist [10]. The protocol and systematic search strategy of the meta-analysis are documented online (PROSPERO registry-CRD42022360304).

### 2.1. Search Strategy and Selection Criteria

We searched PubMed, EMBASE, the Cochrane Library, Web of Science, and OVID from database inception to 13 November 2021, for double-blind randomized controlled trials (RCTs) of extended treatment for DVT, PE, or VTE. The search terms used were ‘anticoagulation’, ‘anticoagulants’, ‘aspirin’, ‘warfarin’, ‘apixaban’, ‘rivaroxaban’, ‘edoxaban’, ‘dabigatran’, ‘randomized controlled trials’, ‘deep venous thrombosis’, ‘pulmonary embolism’, and ‘venous thromboembolism’. To maximize the yield of studies, no language restrictions or further limits were applied. Papers published in a foreign language were translated by the translation tool or data were extracted by relevant foreign language natives if needed. We also reviewed the bibliographies of the relevant articles identified by the search strategy and relevant systematic reviews.

Double-blind RCTs reporting a comparison between any oral anticoagulant or aspirin and another anticoagulant, aspirin, or placebo used for extended anticoagulation for DVT, PE, or VTE were eligible for inclusion. Extended anticoagulation was defined as a duration of anticoagulation treatment > 3 months. We did not limit comorbidities in the population, but studies including disease-specific populations were excluded, such as those with cancer and postsurgical patients. We excluded studies with no information about the duration of follow-up and studies in which the population did not receive initial anticoagulation. Other exclusion criteria included studies with no comparator group, studies in which the definition of the outcome was ambiguous, and studies on unlicensed anticoagulants.

Two investigators (ZQL and YRD) conducted literature searches and determined whether each study was eligible for inclusion independently, and any disagreements were resolved by a consensus decision.

### 2.2. Outcome Assessment

The primary efficacy outcome was the incidence of adjudicated symptomatic recurrent VTE, which was defined as composite DVT or nonfatal or fatal PE. The principal safety outcome was the incidence of adjudicated clinically relevant bleeding, which was defined as a composite of major bleeding or clinically relevant non-major bleeding (CRNMB). According to the International Society of Thrombosis and Haemostasis (ISTH) criteria, major bleeding was defined as overt bleeding that led to a decrease in the hemoglobin level of 2 g per deciliter or more, required transfusion of 2 or more units of red cells, occurred in a critical site, or contributed to the death. CRNMB was defined as overt bleeding that did not meet the criteria for major bleeding but required medical intervention, unscheduled contact with a physician, interruption or discontinuation of the study drug, or discomfort or impairment in performing activities of daily living. The secondary safety outcome was all-cause death.

### 2.3. Data Extraction

Two investigators (ZQL and YRD) independently extracted data from a Microsoft Excel spreadsheet, including the number, type, and definition of outcomes (recurrence, major bleeding, CRNMB, and all-cause death). For all the included studies, we also extracted the following data for each trial, when available: study design, characteristics of the study population (cause of VTE, duration of anticoagulation before randomization, etc.), diagnostic methods, duration of follow-up, anticoagulant type, anticoagulant dose and duration of treatment. Data were extracted for intention-to-treat analyses whenever trial reporting allowed.

### 2.4. Quality Assessment and Risk of Bias

Two investigators (ZQL and YRD) independently conducted risk of bias assessments at the study level using the Cochrane risk of bias tool [11]. Disagreements were resolved by discussion. The methods used to generate the randomization schedule and conceal treatment allocation—whether blinding was implemented for participants, personnel or outcome assessment, whether there was evidence of incomplete outcome data, and whether there was evidence of selective reporting of outcomes—were all recorded in the assessment.

### 2.5. Data Synthesis and Statistical Analysis

We performed a Bayesian NMA using Rstudio (version 4.0.5, R Core Team (2021), Vienna, Austria. URL https://www.R-project.org/) to calculate combined effect values by combining direct and indirect comparisons. Three basic assumptions need to be satisfied to conduct an NMA: homogeneity, similarity, and consistency. We determined the heterogeneity of studies by calculating I^2^ statistics. When I^2^ ≤ 50%, in line with the homogeneity assumption, we constructed a fixed-effect model to calculate effect size; when I^2^ > 50%, there was obvious heterogeneity between studies, and we explored the sources of heterogeneity. When statistical heterogeneity could not be explained, a random-effects model was established to calculate an effect size.

We plotted a network of interventions, with line thickness representing sample size. Sensitivity analysis was carried out by a variety of methods. Trajectory diagrams, density diagrams, and Brooks–Gelman–Rubin diagnosis diagrams were drawn, and the potential scale reduction factor (PSRF) was calculated to judge the convergence of the model. When the trajectory diagram iterations reached more than 5000 iterations, Markov chain Monte Carlo (MCMC) fluctuation was stable and had good overlap. In general, the bandwidth of the density diagram tends to approach 0 and becomes stable when the number of iterations reaches 20,000, and the model has good convergence when the PSRF simultaneously satisfies the following three conditions: (1) the median value of the PSRF tends to approach 1 after n iterations and reaches stability; (2) 97.5% of the PSRF tends to approach 1 and becomes stable after n iterations; and (3) the PSRF value tends to approach 1. HR values and 95% CIs were calculated to assess the efficacy and safety of different anticoagulants, and the different interventions were ranked individually and comprehensively. Finally, the nodal analysis method was used to test the consistency of some comparison results. When *p* values between direct comparison, indirect comparison, and network comparison results were greater than 0.05, we considered that there was no significant difference; that is, the consistency was excellent.

## 3. Results

### 3.1. Study Selection

**Figure 1:**
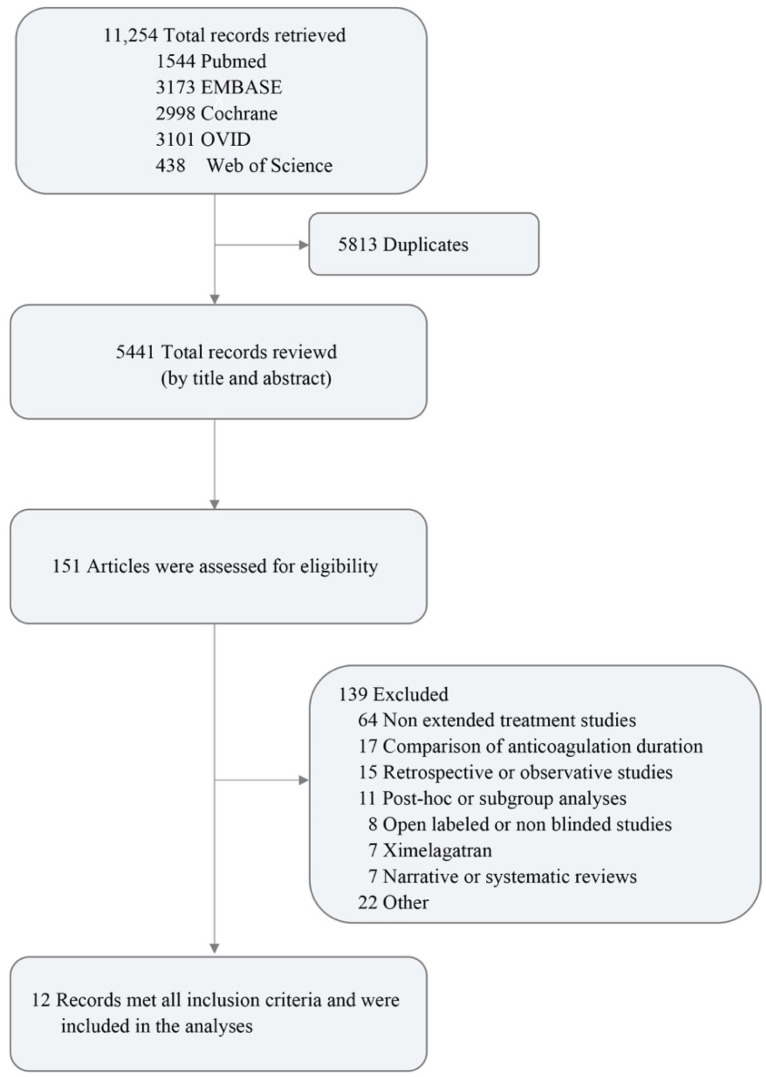
Study Selection Flow Chart.

**Figure 2:**
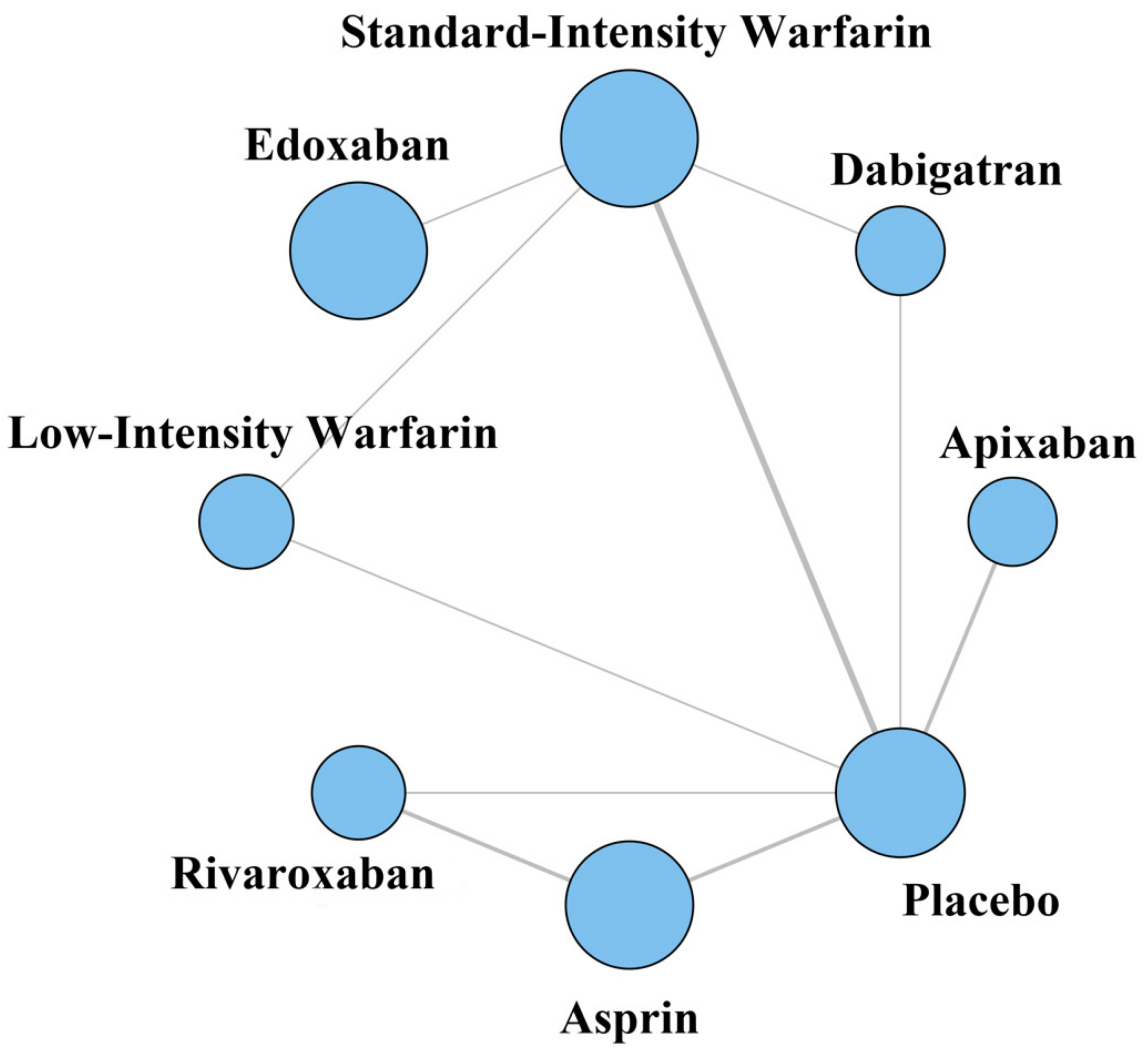
Evidence Network for Included Randomized Controlled Trials. The width of the line for each connection in the evidence network is proportional to the number of randomized controlled trials comparing each pair of treatments. The size of each treatment node is proportional to the patient years of follow-up.

A total of 11,254 studies were retrieved, and 12 articles containing 13 RCTs (21,576 patients) were included in the analyses: PADIS-DVT [5], PADIS-PE [6], PREVENT [12], ASPIRE [13], EINSTEIN-Extension [14], EINSTEIN CHOICE [15], LAFIT [16], WARFASA [17], ELATE [18], RE-SONATE [19], RE-MEDY [19], Hokusai-VTE [20] and AMPLIFY-EXT [21]. The flow chart is presented in Figure 1. The intervention network and comparisons are presented in Figure 2. The baseline characteristics of the included studies are shown in Table 1.

### 3.2. Study Characteristics

The studies included in the network meta-analysis were double-blind RCTs with populations who received more than 3 months of anticoagulation therapy, and there was clinical equipoise regarding the continuation or cessation of anticoagulation therapy. The type of VTE was not limited, and both provoked and unprovoked VTE patients may have been included in some of the studies. The follow-up period in all the studies except RE-SONATE was more than 1 year, and the treatment duration was basically consistent with the follow-up period. Finally, we included 15 data sets from 12 studies with follow-up periods ranging from 0.5 to 3.1 years, with an average follow-up period of 1.45 years and a median follow-up period of 1.08 years. The number of person-years ranged from 66 to 3694, with an average of 961 and a median of 882 person-years. Among the participants, patients with Hokusai-VTE did not receive anticoagulant treatment before recruitment, and patients were followed for 1 year after anticoagulant treatment to assess outcomes. To minimize the heterogeneity between studies, we deleted the data from the first 3 months and analyzed only the data collected between 3 and 12 months after the start of anticoagulant therapy.

The definition of recurrence was consistent across all the studies, and standards of major bleeding were consistent with those defined by the ISTH, but there was no clear definition of CRNMB or minor bleeding in some studies. We only extracted data with a clear definition of CRNMB, and there were too few studies with minor bleeding data, thus no relevant analysis was conducted.

### 3.3. NMA Results

**Figure 3:**
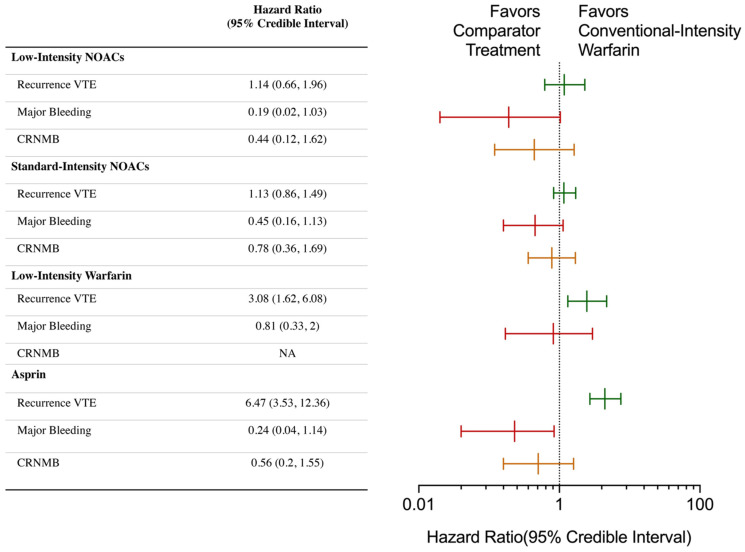
Category network meta-analysis comparing standard-intensity warfarin for the treatment of recurrent venous thromboembolism, major bleeding and clinically relevant non-major bleeding. Hazard ratios greater than 1 favor the treatment group listed in the treatment column; hazard ratios less than 1 favor the comparison treatment. A random-effects model was used because heterogeneity existed between the included studies. Abbreviations: CRNMB, clinically relevant non-major bleeding; NA: not assessed.

**Figure 4:**
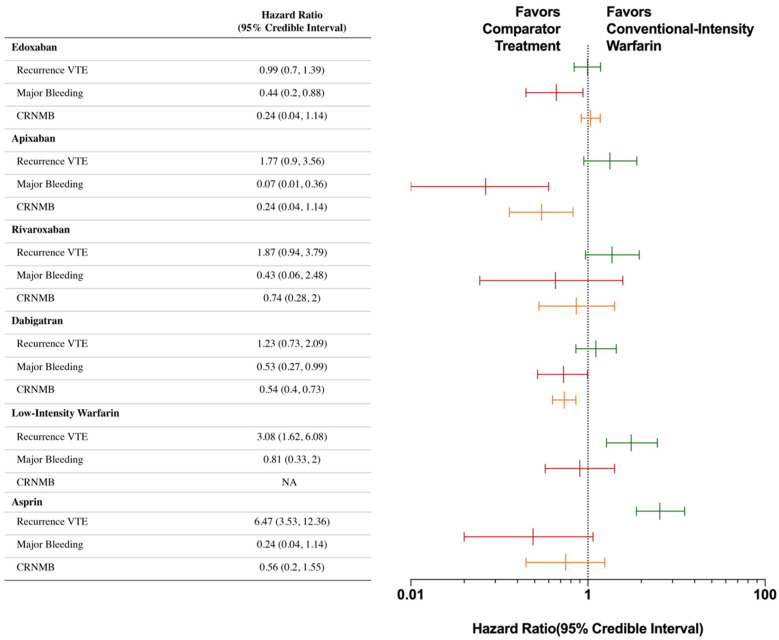
Individual network meta-analysis comparing standard-intensity warfarin for the treatment of recurrent venous thromboembolism, major bleeding and clinically relevant non-major bleeding. Hazard ratios greater than 1 favor the treatment group listed in the treatment column; hazard ratios less than 1 favor the comparison treatment. A fixed-effect model was used. Abbreviations: CRNMB, clinically relevant non-major bleeding; NA: not assessed.

**Figure 5:**
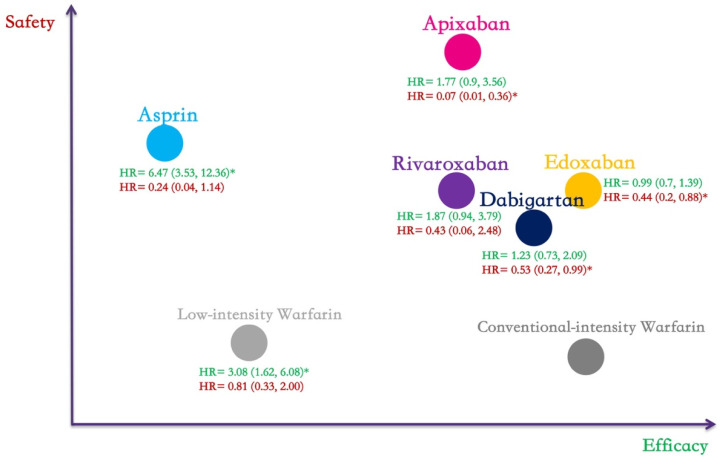
Efficacy (green) and safety (red) comparisons of different anticoagulants in extended anticoagulation therapy for venous thromboembolism. All hazard ratios and 95% confidence intervals were calculated by comparison with standard-intensity warfarin. * Shows a statistically significant difference.

The NMA results of the primary outcomes of different anticoagulants are shown in Figure 3 and Figure 4 and Table 2. Efficacy and safety comparisons of different anticoagulants in extended anticoagulation therapy for venous thromboembolism are shown in Figure 5.

#### 3.3.1. VTE Recurrence

For VTE recurrence, standard-intensity warfarin was associated with the lowest recurrence risk (SUCRA: 0.90) compared to the group of low-intensity NOACs (HR, 1.14, 95% CI: 0.66–1.96, SUCRA: 0.76); the group of standard-intensity NOACs (HR = 1.13, 95% CI: 0.86–1.49, SUCRA: 0.74) were not inferior, and there was no significant difference. Regarding individual drugs, there was no significant difference among the different NOACs, and edoxaban was ranked first among the NOACs (HR = 0.99, 95% CI: 0.70–1.39, SUCRA: 0.88). Dabiatran was ranked second (HR = 1.23, 95% CI: 0.73–2.09, SUCRA: 0.73), apixaban was ranked third (HR = 1.77, 95% CI: 0.90–3.56, SUCRA: 0.54), and rivaroxaban was ranked fourth (HR = 1.87, 95% CI: 0.94–3.79, SUCRA: 0.51). Low-intensity warfarin (HR = 3.06, 95% CI: 1.62–6.03) and aspirin had higher VTE recurrence rates (HR = 6.41, 95% CI: 3.56–12.29) than the NOACs, but the rate of VTE recurrence associated with aspirin was still lower than that with the placebo (HR = 0.72, 95% CI: 0.55–0.93). We performed another analysis using a random-effects model, which showed similar results with wider 95% CIs.

#### 3.3.2. Major Bleeding

For major bleeding, standard-intensity warfarin was associated with the highest major bleeding risk (SUCRA: 0.08) compared with the group of low-intensity NOACs (HR = 0.22, 95% CI: 0.06–0.79, SUCRA: 0.76), and the group of standard-intensity NOACs (HR = 0.46, 95% CI: 0.28–0.73, SUCRA: 0.43) were statistically superior. Regarding individual drugs, apixaban significantly reduced the risk of major bleeding compared with standard-intensity warfarin (HR = 0.07, 95% CI: 0.01–0.37); additionally, it was superior to rivaroxaban (HR = 0.16, 95% CI: 0.03–0.8), edoxaban (HR = 0.16, 95% CI: 0.02–0.98) and dabigatran (HR = 0.13, 95% CI: 0.02–0.75). In addition, compared with standard-intensity warfarin, edoxaban (HR = 0.44, 95% CI: 0.21–0.88, SUCRA: 0.47) and dabigatran were associated with lower risks of major bleeding (HR = 0.84, 95% CI: 0.01–0.37, SUCRA: 0.39). Rivaroxaban was not inferior to standard-intensity warfarin (HR = 0.43, 95% CI: 0.07–2.43, SUCRA: 0.39). Low-intensity warfarin did not reduce the risk of major bleeding (HR = 0.81, 95% CI: 0.32–1.97, SUCRA: 0.19). Aspirin was associated with a lower risk than standard-intensity warfarin, but the difference was not statistically significant. (HR = 0.24, 95% CI: 0.04–1.10, SUCRA: 0.64) We performed another analysis using a random-effects model, which showed wider 95% CIs, and only the comparison between apixaban and standard-intensity warfarin showed a statistically significant difference.

#### 3.3.3. Clinically Relevant Non-Major Bleeding

For non-major bleeding, standard-intensity warfarin was associated with the highest major bleeding risk (SUCRA: 0.006) compared with the group of low-intensity NOACs (HR = 0.47, 95% CI: 0.27–0.84, SUCRA: 0.76), and standard-intensity NOACs (HR = 0.81, 95% CI: 0.67–0.98, SUCRA: 0.26) were statistically superior. Apixaban had similar effects on control of major bleeding and was significantly superior to standard-intensity warfarin (HR = 0.3, 95% CI: 0.13–0.67), edoxaban (HR = 0.28, 95% CI: 0.11–0.64) and rivaroxaban (HR = 0.4, 95% CI: 0.18–0.85), but there was no significant difference when compared with dabigatran (HR = 0.54, 95% CI: 0.24–1.16). Compared with standard-intensity warfarin, dabigatran was associated with a lower risk of clinically related non-major bleeding (HR = 0.54, 95% CI: 0.4–0.73), but edoxaban (HR = 1.08, 95% CI: 0.84–1.38) and rivaroxaban (HR = 0.74, 95% CI: 0.28–2.01) showed no significant differences. Data on low-intensity warfarin are lacking. Aspirin was associated with a lower risk than standard-intensity warfarin, but the difference was not statistically significant (HR = 0.56, 95% CI: 0.20–1.50, SUCRA: 0.54). We performed another analysis using a random-effects model, which showed wider 95% CIs, and all the comparisons showed no statistically significant differences.

#### 3.3.4. All-Cause Death

There was no significant difference in all-cause death. The group of low-intensity NOACs ranked first and was associated with a lower risk of death than standard-intensity warfarin, but the difference was not significant difference (HR = 0.72, 95% CI: 0.27–1.80, SUCRA: 0.88), and standard-intensity warfarin ranked second (SUCRA: 0.72). Apixaban ranked first (HR = 0.71, 95% CI: 0.23–2.16, SUCRA: 0.82), dabigatran ranked second (HR = 0.84, 95% CI: 0.43–1.63, SUCRA: 0.73) and standard-intensity warfarin (SUCRA: 0.63) ranked third. We performed another analysis using a random-effects model, which showed similar results with wider 95% CIs.

### 3.4. Sensitivity Analysis

The results of the heterogeneity test were shown in the Supplementary Figure S1. Most comparators confirmed the homogeneity hypothesis, and a fixed-effect model was constructed to calculate the effect values. Some comparators showed heterogeneity; therefore, a random-effects model was constructed: major bleeding and death evaluations of standard-intensity NOACs. The trajectory diagram, density diagram, Brooks–Gelman–Rubin diagnostic diagram and PSRF all showed that the model converged well, relevant results are shown in Supplementary Figures S2 and S3. All the results conformed to the consistency assumption except for those obtained from the major bleeding evaluation for individual NOACs, relevant results are shown in Supplementary Figure S4.

## 4. Discussion

This NMA included approximately 22,000 patients and assessed the clinical outcomes and safety of prolonged treatment with different anticoagulants for VTE. We provide estimates of the risks of recurrence of symptomatic VTE, clinically relevant bleeding (including major bleeding and CRNMB) and all-cause death, which are clinically relevant and are the outcomes that clinical practice guideline recommendations are based on. For all therapeutic options, standard-intensity warfarin was used as the comparator in the NMA because it is a main component of anticoagulation therapy.

Standard-intensity warfarin was shown to be the most effective in preventing the recurrence of VTE but was accompanied by the highest clinically relevant bleeding risk. It was shown to be excellent in preventing all-cause death. Prolonged use of standard-intensity warfarin, an effective and common anticoagulant, for VTE is associated with a high bleeding risk, a short time in therapeutic range (TTR) and a low rate of INR 2.0–3.0. Hence, there is a need for frequent INR monitoring, which leads to low patient compliance. Low-intensity warfarin did not reduce the risk of clinically relevant bleeding but was associated with a statistically significant increase in VTE recurrence; moreover, due to the risk of all-cause death, a reduced dose of warfarin is not recommended to prevent VTE recurrence.

NOACs effectively overcome the shortcomings of warfarin and can potentially be used in extended treatment for VTE. NOACs were not inferior to standard-intensity warfarin in terms of VTE recurrence and were superior in preventing clinically relevant bleeding. Although the difference was not statistically significant, low-intensity NOACs seem to be superior to standard-intensity NOACs in terms of both recurrence and clinically relevant bleeding. This may be because patients using low-intensity NOACs may be more likely to achieve therapeutic levels and receive thorough follow-up and dosing guidance. In individuals, edoxaban was the most effective NOAC in preventing VTE recurrence and ranked second only to apixaban in reducing the risk of major bleeding, indicating that it is an effective and safe anticoagulant for extended treatment of VTE. Apixaban was not inferior to standard-intensity warfarin in terms of the recurrence of VTE and had a significant advantage in controlling clinically relevant bleeding. It showed significant superiority over standard-intensity warfarin and the other three NOACs in controlling major bleeding and CRNMB, apart from the fact that no statistically significant difference was observed in the control of CRNMB when compared with dabigatran, which suggests that apixaban is the ideal anticoagulant in patients at high risk of bleeding. Dabigatran was not inferior to standard-intensity warfarin in terms of the recurrence of VTE and demonstrated a good ability to control clinically relevant bleeding; it was ranked second to only apixaban in reducing the risk of CRNMB. Rivaroxaban was not inferior to standard-intensity warfarin in terms of recurrence of VTE, but NOACs alone did not show statistical superiority.

Aspirin was less effective than standard-intensity warfarin but was still superior to the placebo; it was not associated with an increased risk of major bleeding, but it was associated with an increased risk of CRNMB compared to the placebo. Aspirin should still be considered in patients who cannot receive anticoagulant therapy.

In initial treatment of VTE, anticoagulation or thrombolytic treatment were selected according to the risk stratification, a study revealed that anticoagulation before thrombolytic therapy was safer than anticoagulation following thrombolytic therapy in systemic thrombolytic therapy for PE [22]. Low-molecular-weight heparin (LMWH) or VKAs are the recommended anticoagulation therapies for VTE; however, rivaroxaban and apixaban were associated with the lowest risks of bleeding [23]. Considering that there was no comparator for edoxaban or dabigatran in the NMA for initial treatment of VTE, the NMA of extended treatment of VTE was basically consistent with the conclusion of the former. Additionally, our study was consistent with a meta-analysis that compared NOACs to VKAs in patients with VTE, including those with thrombophilia, the finding revealed rates of VTE recurrence and clinically relevant bleeding events were both low and comparable in patients with various thrombophilias receiving either treatment [24]. Clinicians should have more confidence in using NOACs for VTE when assessing the benefit–harm balance of the various treatment options and tailoring their therapeutic approaches accordingly.

Our findings are consistent with the results of two prior published systematic reviews and meta-analyses on the extended use of oral anticoagulants for the secondary prevention of VTE [25,26]. Compared with these prior reports, we were more rigorous in determining whether studies were included in our review, and we did not include the DURAC II, WODIT-DVT [27] and WODIT-PE [28] open-label trials, which allowed us to better control heterogeneity. We include the important data of edoxaban in the network. We calculated the clinical outcomes and safety of the collective set of NOACs and different individual NOACs. By examining the clinical outcomes associated with and the safety of each individual NOAC, the clinical outcomes associated with and safety of NOACs as a whole may have been overshadowed; for example, apixaban was effective in controlling clinically relevant bleeding. All results were analyzed with both the fixed-effect model and the random-effects model; both effects models showed consistent results, and the random-effects model had a wider CI.

It is important to note the limitations of our study. First, we did not limit the type of VTE in the study, which may have led to potential heterogeneity. Studies have shown that the risk of recurrence of provoked VTE is lower than that of unprovoked VTE [29]. Second, although we included only studies in which the population received initial anticoagulant treatment for more than 3 months, the duration of initial anticoagulant treatment varied, and the cumulative recurrence rate of VTE increased as the duration of anticoagulant treatment increased. Third, we did not include many studies, which limited our ability to build a comprehensive network with high credibility. Fourth, the results of the effects of individual NOACs on major bleeding were not consistent because we obtained only results of indirect comparisons, and there was an insufficient number of studies to build a credible network. Fifth, RE-MEDY did not provide median or mean follow-up times, and we used the follow-up time of the study design. Sixth, death was caused by multiple factors, and anticoagulant use alone may not have directly led to death, so the factors leading to all-cause death should be assessed more cautiously.

## 5. Conclusions

By pooling different anticoagulants used for prolonged treatment of VTE and conducting a meta-analysis, we found that there was no statistically significant difference in recurrence associated with NOACs when compared with standard-intensity warfarin, and NOACs were associated with a lower risk of bleeding. Edoxaban was effective in preventing both recurrence and major bleeding, and apixaban was the best anticoagulant for control of clinically relevant bleeding.

## Figures and Tables

**Table 1:** Baseline characteristics of randomized clinical trials of extended treatment of venous thromboembolism comparing oral anticoagulant or aspirin with another anticoagulant, aspirin or placebo.

Source	Sample Size	Duration of Follow Up, Years ^a^	Recurrent Venous Thromboembolism Events	Major Bleeding	CRNMB	All-Cause Death
**LAFIT, 1999 [16]**						
Warfarin-S	79	0.83	1	0	None	1
Placebo	83		17	3	None	3
**PREVENT, 2003 [12]**						
Warfarin-L	255	2.10	14	5	None	4
Placebo	253		37	2	None	8
**ELATE, 2003 [18]**						
Warfarin-L	369	2.40	16	9	None	16
Wafarin-S	369		6	8	None	8
**EINSTEIN–Extension, 2010 [14]**						
Rivaroxaban	602	1.00	8	4	32	1
Placebo	594		42	0	7	2
**ASPIRE, 2012 [13]**						
Asprin	411	3.10	57	8	6	16
Placebo	411		73	6	2	18
**WARFASA, 2012 [17]**						
Asprin	205	2.07	28	1	3	6
Placebo	197		43	1	3	5
**AMPLIFY-EXT, 2012 [21]**						
Apixaban-L	840	1.08	14	2	25	7
Apixaban-S	813		14	1	34	4
Placebo	829		73	4	19	14
**RE-SONATE, 2013 [19]**						
Dabigatran	681	0.50	3	2	34	None
Placebo	662		35	0	12	None
**RE-MEDY, 2013 [19]**						
Dabigatran	1430	1.58	26	13	67	17
Warfarin-S	1426		18	25	120	19
**Hokusai-VTE, 2013 [20]**						
Edoxaban	3633	1.00	66	11	132	51
Warfarin-S	3594		67	24	124	43
**PADIS-PE, 2017 [6]**						
Warfarin-S	184	1.50	28	6	None	13
Placebo	187		39	5	None	6
**EINSTEIN CHOICE, 2017 [15]**						
Rivaroxaban-L	1127	1.00	13	5	22	2
Rivaroxaban-S	1107		17	6	30	8
Asprin	1131		59	3	20	7
**PADIS-DVT, 2019 [5]**						
Warfarin-S	50	1.50	14	1	None	1
Placebo	54		17	0	None	3

Abbreviations: CRNMB, clinically relevant non-major bleeding; S: standard intensity; ^a^ Average or median duration of follow-up used. L: low intensity.

**Table 2:** Treatment comparisons within the network meta-analysis for recurrent venous thromboembolism, major bleeding, CRNMB and all-cause death.

	Hazard Ratios (95% Credible Interval)			
Treatment	Recurrence	Major Bleeding	CRNMB	All-Cause Death
**Compared with Standard-Intensity Warfarin**				
Edoxaban	0.99 (0.7, 1.39)	**0.44 (0.2, 0.88)**	1.07 (0.84, 1.38)	1.18 (0.79, 1.77)
Apixaban	1.77 (0.9, 3.56)	**0.07 (0.01, 0.36)**	**0.3 (0.13, 0.68)**	0.71 (0.23, 2.16)
Rivaroxaban	1.87 (0.94, 3.79)	0.43 (0.06, 2.48)	0.74 (0.28, 2)	1.09 (0.31, 3.86)
Dabigatran	1.23 (0.73, 2.09)	0.53 (0.27, 0.99)	0.54 (0.4, 0.73)	0.84 (0.43, 1.63)
Low-Intensity Warfarin	**3.08 (1.62, 6.08)**	0.81 (0.33, 2)	NA	1.44 (0.7, 3.01)
Aspirin	**6.47 (3.53, 12.36)**	0.24 (0.04, 1.14)	0.56 (0.2, 1.55)	1.64 (0.58, 4.89)
**Compared with Edoxaban**				
Apixaban	1.79 (0.84, 3.91)	**0.16 (0.02, 0.99)**	**0.28 (0.11, 0.65)**	1.64 (0.58, 4.89)
Rivaroxaban	1.9 (0.88, 4.16)	0.99 (0.13, 6.62)	0.69 (0.25, 1.93)	0.92 (0.25, 3.51)
Dabigatran	1.25 (0.67, 2.32)	1.2 (0.45, 3.22)	**0.51 (0.34, 0.74)**	0.71 (0.33, 1.54)
Low-Intensity Warfarin	**3.12 (1.51, 6.65)**	1.86 (0.59, 5.97)	NA	1.22 (0.53, 2.8)
Aspirin	**6.55 (3.25, 13.61)**	0.54 (0.08, 3.12)	0.52 (0.18, 1.48)	1.38 (0.45, 4.4)
**Compared with Apixaban**				
Rivaroxaban	1.06 (0.6, 1.86)	**6.34 (1.26, 35.92)**	**2.49 (1.18, 5.65)**	1.54 (0.51, 4.67)
Dabigatran	0.7 (0.33, 1.46)	**7.7 (1.34, 56.25)**	1.83 (0.86, 4.08)	1.19 (0.33, 4.39)
Low-Intensity Warfarin	1.75 (0.88, 3.42)	**11.97 (2.1, 87.48)**	NA	2.04 (0.68, 6.22)
Aspirin	**3.66 (2.3, 5.93)**	3.49 (0.82, 16.35)	1.89 (0.86, 4.4)	2.32 (0.99, 5.7)
**Compared with Rivaroxaban**				
Dabigatran	0.66 (0.31, 1.39)	1.21 (0.2, 8.47)	0.73 (0.28, 1.87)	0.78 (0.19, 3.19)
Low-Intensity Warfarin	1.65 (0.83, 3.25)	1.89 (0.31, 13.31)	NA	1.32 (0.38, 4.67)
Aspirin	**3.45 (2.44, 5.02)**	0.55 (0.22, 1.31)	0.76 (0.51, 1.12)	1.5 (0.73, 3.24)
**Compared with Dabigatran**				
Low-Intensity Warfarin	**2.5 (1.19, 5.39)**	1.55 (0.52, 4.7)	NA	1.71 (0.64, 4.58)
Aspirin	**5.26 (2.69, 10.67)**	0.45 (0.07, 2.36)	1.03 (0.39, 2.74)	1.94 (0.56, 6.92)
**Compared with Low-Intensity Warfarin**				
Aspirin	**2.1 (1.15, 3.88)**	0.29 (0.05, 1.51)	NA	1.14 (0.4, 3.3)

Abbreviation: CRNMB, clinically relevant non-major bleeding. Values in bold indicate statistical differences. Hazard ratios greater than 1 favor the treatment group listed in the treatment column. A fixed-effect model was used; hazard ratios less than 1 favor the comparison treatment.

## Data Availability

Additional data will be made available to all readers upon request per email.

## Supplementary Materials

The following supporting information can be downloaded at: https://www.mdpi.com/article/10.3390/jcdd9120414/s1. Figure S1: Heterogeneity Test. Figure S2: Brooks-Gelman-Rubin diagnostic diagram. Figure S3: Trajectory Diagram. Figure S4: Consistency Test.

## Abbreviations

DVT, deep venous thrombosis; PE, pulmonary embolism; VTE, venous thromboembolism; RCTs, randomized controlled trial; NOACS, novel oral anticoagulants; CRNMB, clinically relevant non-major bleeding; SUCRA, surface under the cumulative ranking curve.
