# Supplementary material for: Clinical and Safety Outcomes Associated with Extended Treatment of Venous Thromboembolism: A Network Meta-Analysis
Source: J Cardiovasc Dev Dis. 2022 Nov 25;9(12):414. doi: 10.3390/jcdd9120414 (PMC9781418; doi:10.3390/jcdd9120414)
Supplement: Supplementary file 1 [file jcdd-09-00414-s001.zip › jcdd-2024327-supplementary.pdf]

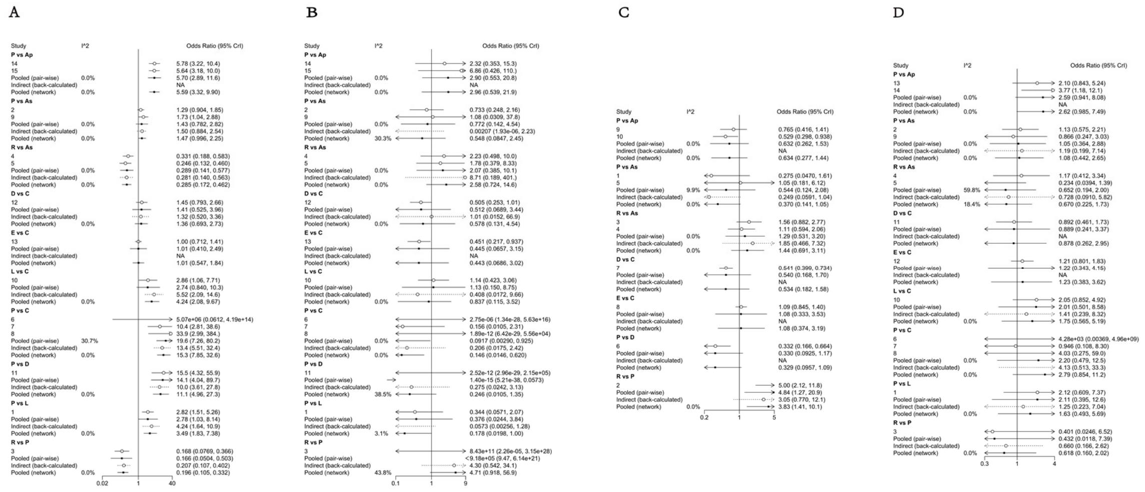

Figure S1 Heterogeneity Test

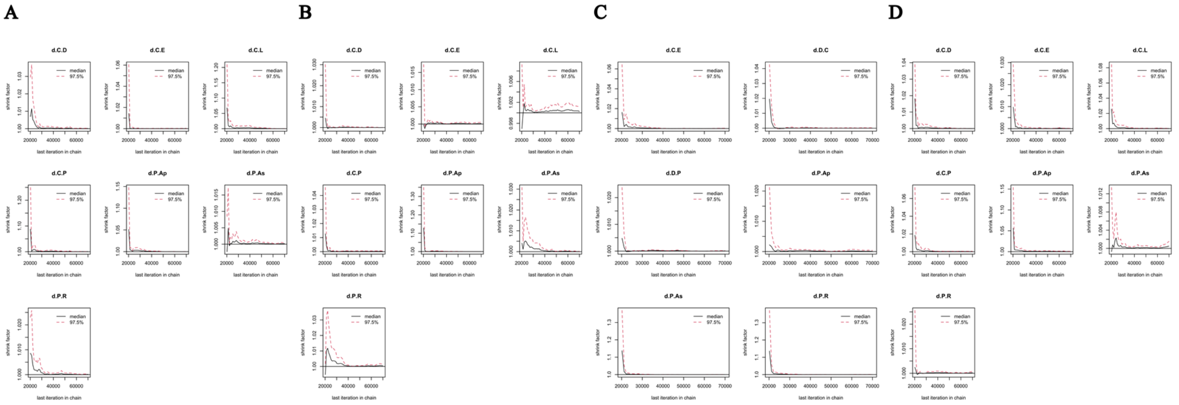

Figure S2 Brooks-Gelman-Rubin diagnostic diagram

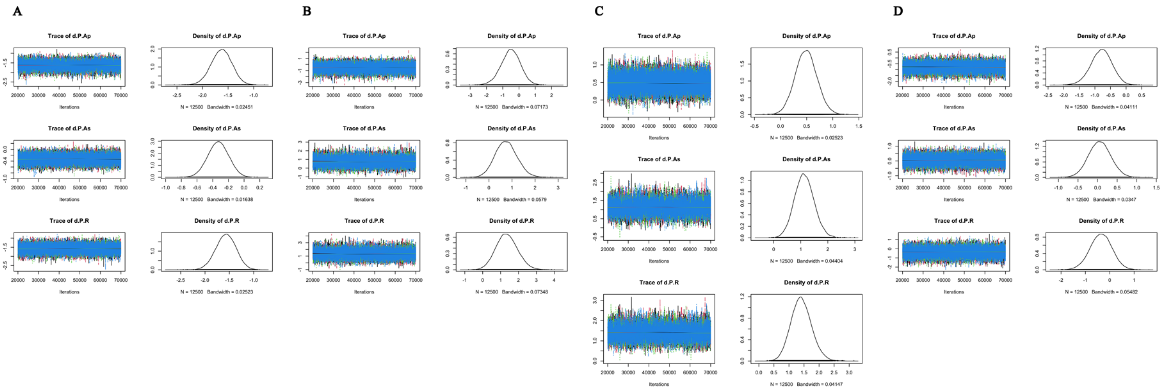

Figure S3 Trajectory Diagram

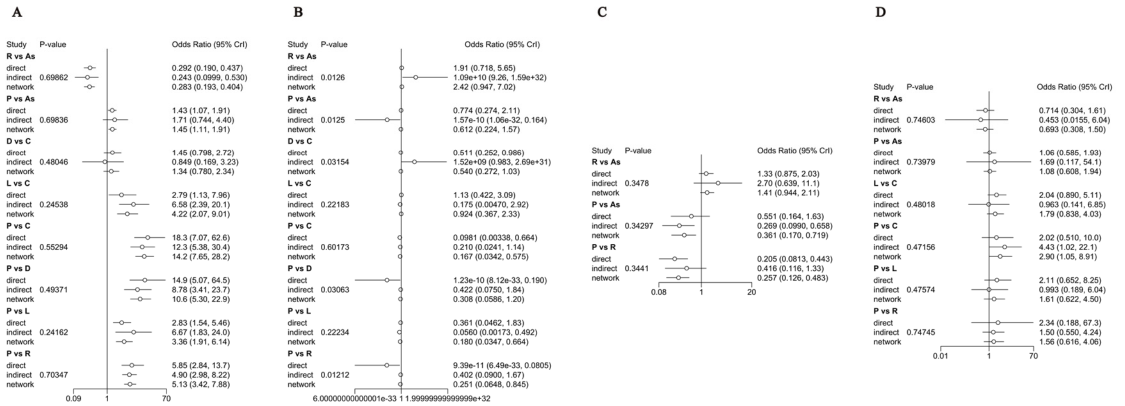

Figure S4 Consistency Test

The letters of the corner mark represent different outcomes:
A, Recurrence;
B, Major Bleeding;
C, Clinically Relevant Non-Major Bleeding;
D, All-cause Death

The letters in the picture stand for different anticoagulants:
Ap, Apixaban;
As, Asprin;
E, Edoxaban;
R, Rivaroaban;
L, Low-intensity Warfarin;
C, Conventional-intensity Warfarin;
P, Placebo;
D, Low-intensity NOACs;
H, Standard-intensity NOACs

The Number in the pircture represent the study include.
